# Nematode parasite assemblages in tusk (*Brosme brosme*) along the Norwegian continental shelf: indicators of fish host feeding ecology and stock structuring

**DOI:** 10.1186/s13071-026-07535-6

**Published:** 2026-07-08

**Authors:** Paolo Cipriani, Miguel Bao, Lucilla Giulietti, Julia E. Storesund, Kristin Helle, Arne Levsen

**Affiliations:** Institute of Marine Research, Bergen, Norway

**Keywords:** *Anisakis*, *Contracaecum*, *Phocanema*, Bioindicators, Seafood safety, Zoonotic parasite

## Abstract

**Background:**

Tusk (*Brosme brosme*) is a demersal species of increasing commercial importance in Norwegian fisheries, yet information on its parasite fauna remains limited. This study represents the first systematic investigation of ascaridoid nematodes infecting tusk from six localities across a wide latitudinal gradient in Norwegian waters, spanning from the North Sea to the Barents Sea.

**Methods:**

A total of 187 fish from commercial catches were examined using the UV press method, and 225 anisakid larvae were genetically identified through mitochondrial DNA (cox2) sequencing.

**Results:**

Nine species were recorded: *Anisakis simplex* (s.s.), *Phocanema decipiens* (s.s.), *P. krabbei*, *P. bulbosum*, *Contracaecum osculatum* B, *Phocascaris cystophorae*, *Phocascaris* sp., *Hysterothylacium aduncum*, and *Hysterothylacium* sp. *Anisakis simplex* (s.s.) occurred at 100% prevalence and was detected in all examined tissues, including viscera, muscle, liver, and gonads. Notably, 42% of larvae were located in the musculature, with 92% of flesh invading larvae concentrated in the belly flap region. *Phocanema* spp. were also consistently present, with *P. decipiens* (s.s.) and *P. krabbei* predominantly infecting muscle tissue, whereas *Contracaecum*, *Phocascaris*, and *Hysterothylacium* spp. were restricted to the viscera. Distinct geographic patterns were observed among *Phocanema* species, including the occurrence of *P. bulbosum* in Arctic samples.

**Conclusions:**

Spatial differences in parasite assemblages between northern and southern sampling areas suggest ecological structuring, likely reflecting variation in definitive host distribution and trophic interactions, and support the use of anisakid parasites as biological tags for supplemental stock discrimination. The observed infection patterns further suggest that tusk trophic ecology may involve a stronger piscivorous component than previously assumed. High infection levels and the frequent occurrence of zoonotic anisakids in the musculature highlight potential food safety and product quality concerns.

**Graphical Abstract:**

**Figure:**
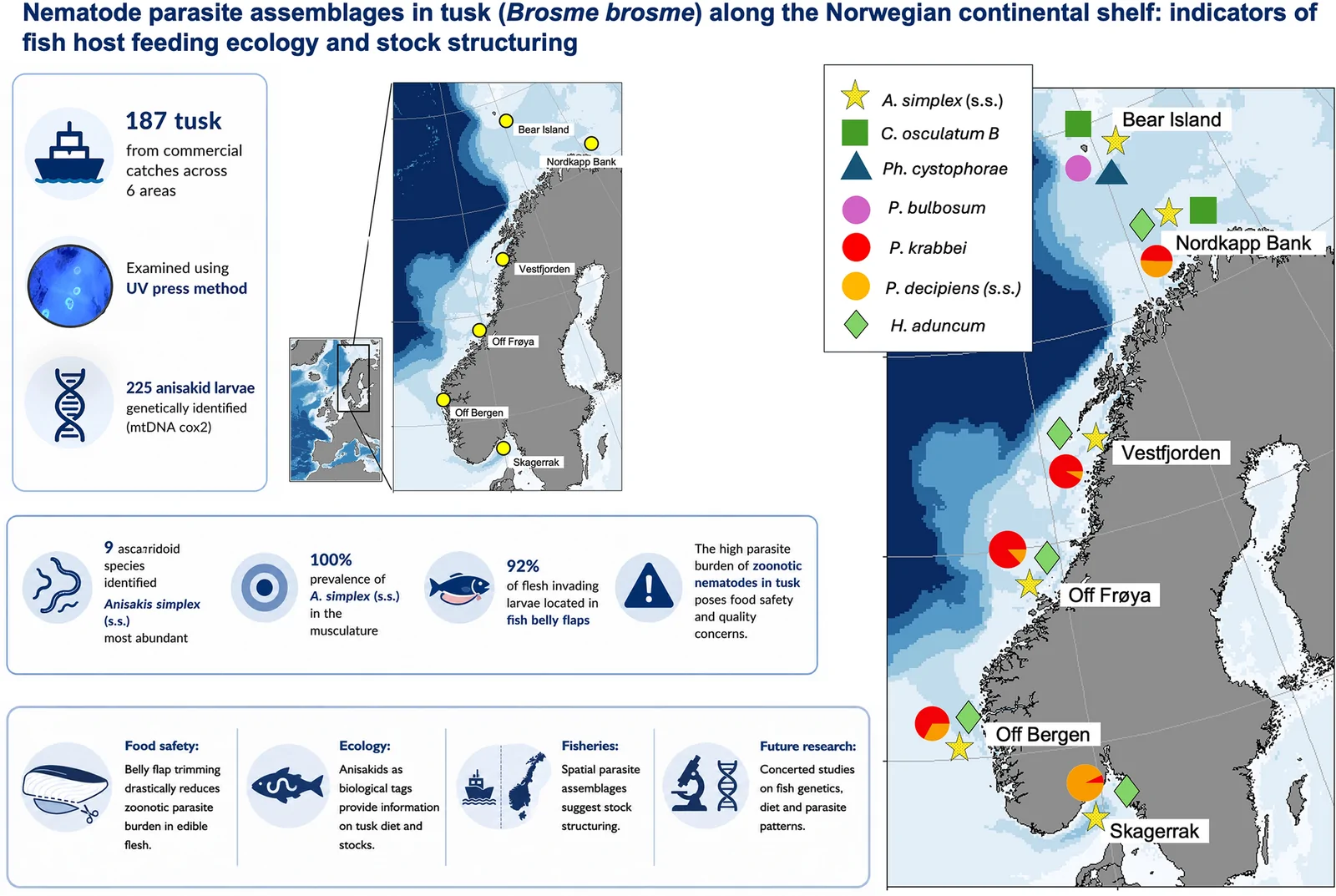

## Background

The tusk, *Brosme brosme* (Ascanius 1772), also known as cusk in the Northwest Atlantic, is a demersal gadiform fish widely distributed in the North Atlantic. It inhabits rocky and muddy substrates at depths ranging from 50 to 1000 m, with a preference for waters between 200 and 500 m [1, 2]. In the Northeast (NE) Atlantic, tusk is found from the Bay of Biscay northwards to Norway, Iceland, the Faroe Islands, and the Barents Sea [2, 3]. It is a generalist predator, feeding primarily on crustaceans, polychaetes, cephalopods, and various fish species [1, 4]. In Norwegian waters, tusk spawns between April and July, with peak spawning occurring at depths of 200–500 m and water temperatures of 4–8 °C [5]. Tusk eggs and larvae are pelagic, while juveniles transition to a demersal lifestyle at approximately 6 cm in length [6]. During the pelagic stage, eggs, larvae, and fry can disperse over considerable distances [7]. Tusk is a commercially important fishery resource in the NE Atlantic and Arctic waters. In 2024, a total of 17,600 tons were landed, of which Norway accounted for 13,300 tons, approximately 76% of the total. In the Barents Sea and the Norwegian Sea, the most important areas for the Norwegian tusk fishery, 9300 tons were landed in 2024 [8]. Despite its commercial importance, several aspects of tusk ecology, feeding behaviour, and possible population structuring across NE Atlantic waters remain poorly understood.

Ascaridoid nematodes are a diverse group of marine parasites with complex life cycles. For anisakids, cetaceans serve as the primary definitive hosts for genera such as *Anisakis*, *Skrjabinisakis*, and *Pseudoterranova*, while pinnipeds predominantly host adult *Contracaecum, Phocanema* and *Phocascaris*. In contrast, adults of the raphidascaridid nematode *Hysterothylacium* spp. occur in fish. All these nematodes also involve a wide range of intermediate and transport hosts, including small crustaceans as intermediate hosts, followed by fish and cephalopods, which serve as intermediate or paratenic hosts [9]. Because transmission occurs through predation, anisakid assemblages in fish may reflect host diet, habitat use, and exposure to different marine mammal communities, and have therefore been used as biological indicators in studies of trophic ecology, host movements, and stock structure [10–12]. Several anisakid species are also of concern for seafood safety and quality, as larvae present in edible tissues may cause anisakidosis in humans or reduce product quality and marketability [13–15]. Although infection levels and characteristics of ascaridoid nematode larvae have been studied in many commercially important fish species from the Northeast Atlantic [12, 16, 17], information on the occurrence of these parasites in tusk is scarce and fragmented [18]. Reported a 100% prevalence of *Anisakis simplex* (s.l.) in eight tusk specimens from Vestland (southwest Norway) and identified *Phocanema decipiens* (reported as *Porrocaecum decipiens*) in the musculature, as well as the raphidascaridid nematode *Hysterothylacium aduncum* (reported as *Contracaecum aduncum*) and *Contracaecum* s.l. in the viscera. A 100% prevalence of *A. simplex* (s.l.) and the presence of *H. aduncum* were also reported in eight tusk specimens from the deep waters off the Faroe Islands [19]. Several *A. simplex* s.l. larvae were identified in tusk specimens from the North Sea and other Northeast Atlantic areas [20, 21]. In the Gulf of Maine, Nova Scotia, *P. decipiens* was found with 100% prevalence in 12 tusk specimens, with an abundance of 29 and a density of 11 larvae per kilogram of whole fish [22, 23] examined over 100 tusk specimens from Icelandic waters, recording high infection levels of *P. decipiens* (s.l.) and *Anisakis* sp. in the musculature. The most recent study reported 100% prevalence of *A. simplex* (s.s.) in tusk (*N* = 4) from the FAO 27 area [24].

The present study aims to characterize ascaridoid parasite assemblages in tusk from Norwegian waters across a broad latitudinal gradient from Skagerrak to Bear Island. Parasite species diversity, tissue distribution, and spatial variation in infection patterns are analysed to provide insights into tusk trophic ecology and possible population structuring. In addition, the occurrence of anisakid larvae in edible tissues is examined to establish baseline information on their potential relevance for seafood quality and safety in this species.

## Methods

### Fish sampling

**Fig. 1:**
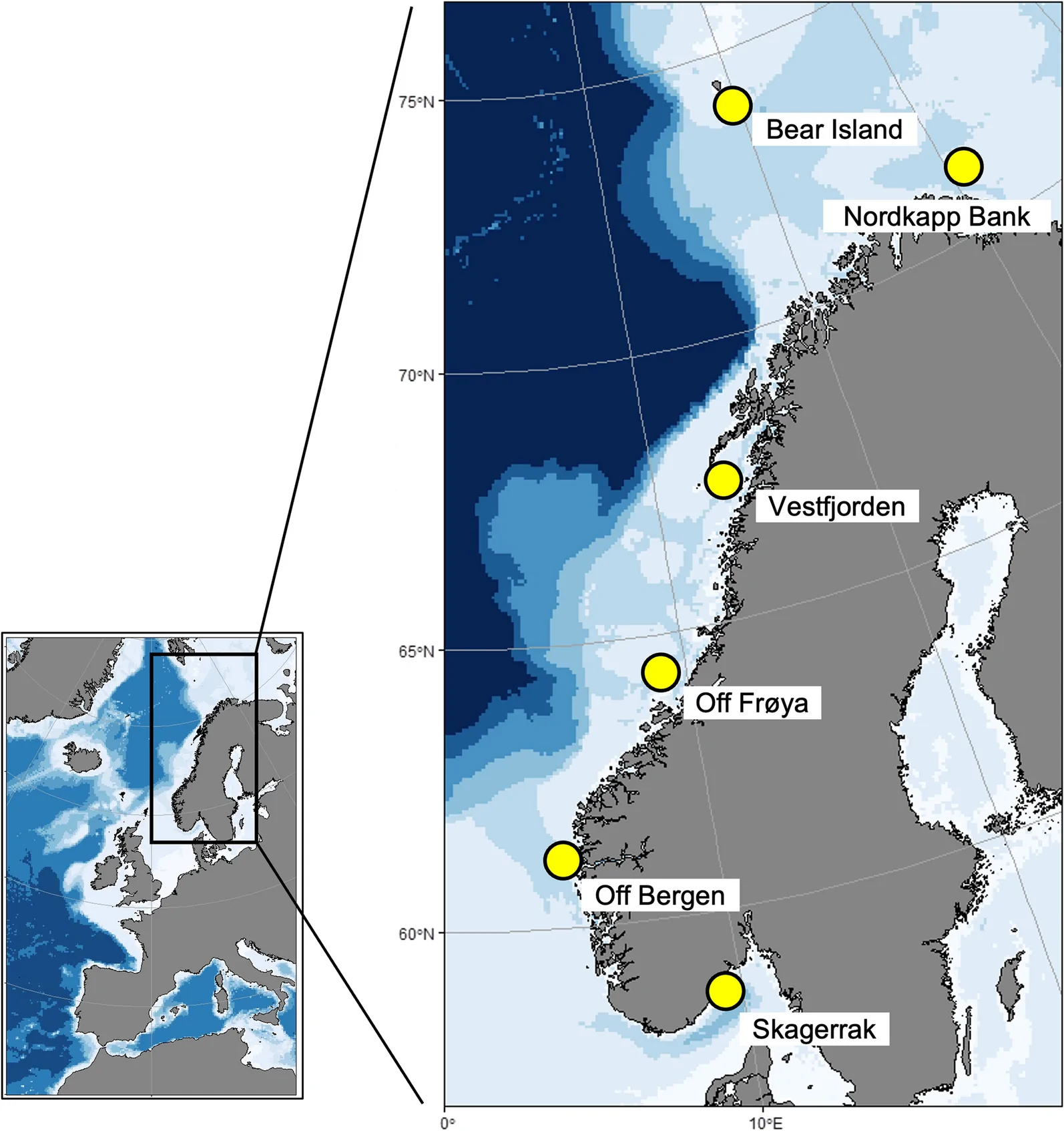
Map illustrating the sampling locations of tusk (*Brosme brosme*)

A total of 187 tusk specimens were collected from sampling areas along the Norwegian continental shelf covering a broad latitudinal gradient from Skagerrak to Bear Island in the Barents Sea. The fish originated from commercial catches obtained from local fishermen and research cruises conducted between 2020 and 2025. Fish were captured using longlines and trawl nets. The catch locations and dates, number of fish examined, and biometric data are shown in Fig. 1 and Table 1. After capture, fish were immediately frozen (approximately 20 °C) and sent to the laboratory of the Institute of Marine Research (IMR), Bergen, Norway, for parasitological inspection.

**Table 1:** Summary of sampling information and biometric data of *Brosme brosme* examined in this study across six Norwegian fishing areas

	*N*	Month–year of catch	Coordinates	ML (cm) ± SD (min max)	MW (kg) ± SD
Bear Island	30	February 2025	74.38721°, 19.35380°	54.0 ± 8.3 (42.0–75.5)	1.9 ± 1.2
Nordkapp Bank	30	November 2023	70.98333°, 31.00000°	49.2 ± 4.4 (44.0–59.0)	1.4 ± 0.4
Vestfjorden	31	October 2023	68.00000°, 14.03000°	56.5 ± 5.6 (31.5–66.0)	1.9 ± 0.4
Off Frøya Isl	33	September 2023	63.73124°, 9.16128°	53.2 ± 9.4 (40.0–73.0)	1.9 ± 1.1
Off Bergen	32	December 2020	60.62708°, 4.90168°	73.8 ± 8.5 (59.0–91.0)	4.6 ± 1.6
Skagerrak	31	May 2024	58.24653°, 8.63118°	75.4 ± 11.0 (43.5–91.0)	4.7 ± 1.8

For each sampling location, the number of fish analysed (*N*), date of catch, geographic coordinates (decimal degrees), mean total length (ML, cm ± SD; range in parentheses), and mean body weight (MW, kg ± SD) are reported

### Parasitological inspection and morphological identification

After thawing, each fish was measured (total body length—TL), weighed (total body weight—TW), and sex was determined. Additionally, liver, gonad and fillet weights were recorded. The data were tested for normality and homogeneity of variance among samples using Shapiro–Wilk and Levene’s tests, respectively. Differences in host biometric parameters such as TL, TW between sampling areas were assessed using Kruskal–Wallis ANOVA followed by post hoc multiple comparisons of significance. All statistical analyses were performed using Statistica^®^ 13.5.0.17 (TIBCO Software Inc., California, USA).

The fish were initially subjected to a gross visual examination for the presence of anisakid nematodes. Fish were cut open, and the visceral cavity and organs were macroscopically examined for presence of the presence of anisakid larvae. Fish fillets were also visually inspected. Subsequently, the viscera and each fillet were individually placed in separate transparent plastic bags for anisakid detection using the UV-press method (ISO 23036–1:2021). The method was used for detection and quantification of larvae in fish tissues (i.e. musculature and visceral organs), and for preliminary differentiation of larvae belonging to different ascaridoids genera *Anisakis*, *Contracaecum*, *Phocanema, Phocascaris* and *Hysterothylacium* based on differences in shape, brightness, coloration, and fluorescence intensity [12, 25]. This initial visual screening was followed by microscopic morphological analysis of anisakid larvae to assign the worms to the different genera according to the diagnostic characters provided by [18, 26]. These characters included the position of the excretory pore relative to the boring tooth and nerve ring, the shape and length of the ventricle, the presence or absence of an intestinal caecum, a ventricular appendix, a terminal mucron, and cuticle ornamentation [18, 26].

### Genetic identification

A subsample of 225 anisakid larvae representing all genera, collected from various infection sites (liver, pyloric caeca, other visceral tissues, and skeletal musculature), representative of all geographical areas, was then genetically identified using mitochondrial cytochrome c oxidase II (mtDNA *cox*2) gene sequencing. DNA extraction was performed using the DNeasy Blood & Tissue Kit (Qiagen), following the manufacturer’s protocol. Amplification of the *cox2* gene was carried out using the primers 211F (5′-TTTTCTAGTTATATAGATTGRTTTYAT-3′) and 210R (5′-CACCAACTCTTAAAATTATC-3′) [27]. Polymerase chain reaction (PCR) was conducted following the protocol described by [28], under the following thermal conditions: initial denaturation at 94 °C for 5 min, followed by 35 cycles of 94 °C for 30 s, 55 °C for 60 s, and 72 °C for 90 s, with a final extension step at 72 °C for 10 min. PCR products were purified and sequenced by Eurofins (Cologne, Germany). Sequence assembly was performed using ChromasPro 2.1.5 (Technelysium Pty Ltd., Tewantin, Australia), and sequence analysis was conducted using the GenBank database (BLAST, http://www.ncbi.nlm.nih.gov/BLAST).

### Infection descriptors and statistics

Quantitative descriptors of anisakid infection parameters were calculated following [29] and included prevalence (*P*, %), abundance (A), mean intensity (mI) ± standard deviation (SD), range (min–max), and density (number of larvae per 100 g fish flesh). These parameters were assessed for different anatomical sites, including the liver, gonads, other visceral organs (including larvae loose in the visceral cavity), and the body musculature. The latter was further subdivided into four sections per flesh side (anterior ventral, posterior ventral, anterior dorsal and posterior dorsal) following [12].

Possible differences in anisakid infection levels between the sampling localities of tusk were assessed using Kruskal–Wallis ANOVA followed by post hoc comparison of significance. The relationships between host body size and *Anisakis* larval intensities were analysed with Spearman’s rank correlation test. Generalized linear models (GLM) were fitted to specifically assess the effect of catching locality and host size (weight) on overall larval intensity and in the flesh, separately for the two most abundant species *A. simplex* (s.s.) and *P. decipiens* (s.l.). Prior to analysis, host weight (TW) and intensity data were log-transformed (LOG (*N* + 1) to better fit a normal log-link distribution. In both models, catching locality was treated as a grouping factor while fish weight (TW) was used as a continuous predictor. The goodness of fit of each model was accepted when the ratio deviance/df was ≤ 1. The significance of the effect of fish weight and locality on larval intensities was assessed with a likelihood type 3 test.

## Results

**Fig. 2:**
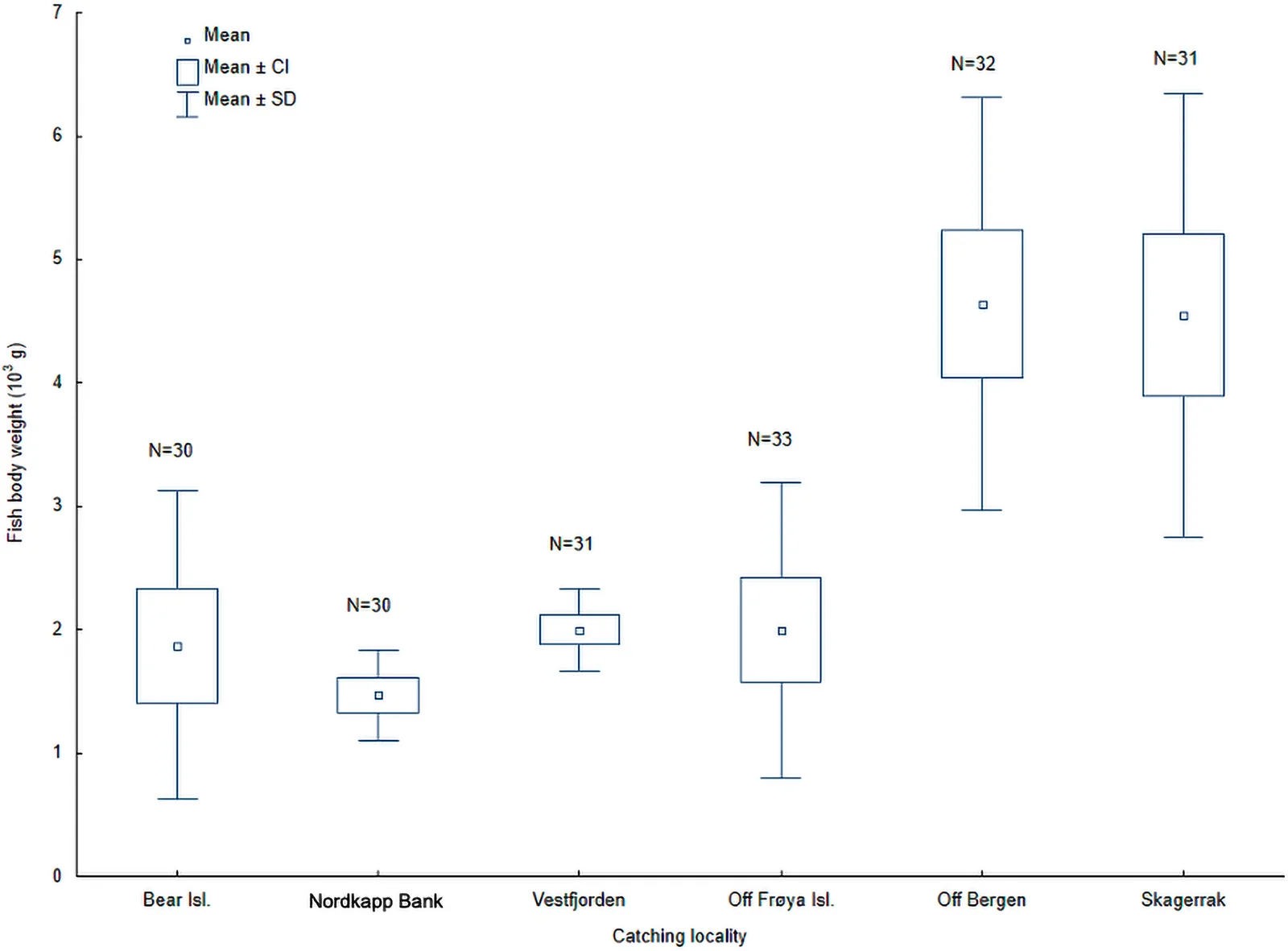
Fish body weight by catching locality of tusk

All fish examined in this study were of commercial size (> 40 cm), but differed significantly in body size (TW) among sampling areas, with larger individuals caught in the two southern localities compared with fish from the central and northern catching areas (Kruskal–Wallis ANOVA followed by post hoc multiple comparisons of significance, *p* < 0.0001) (Fig. 2).

### Parasite identification

**Fig. 3:**
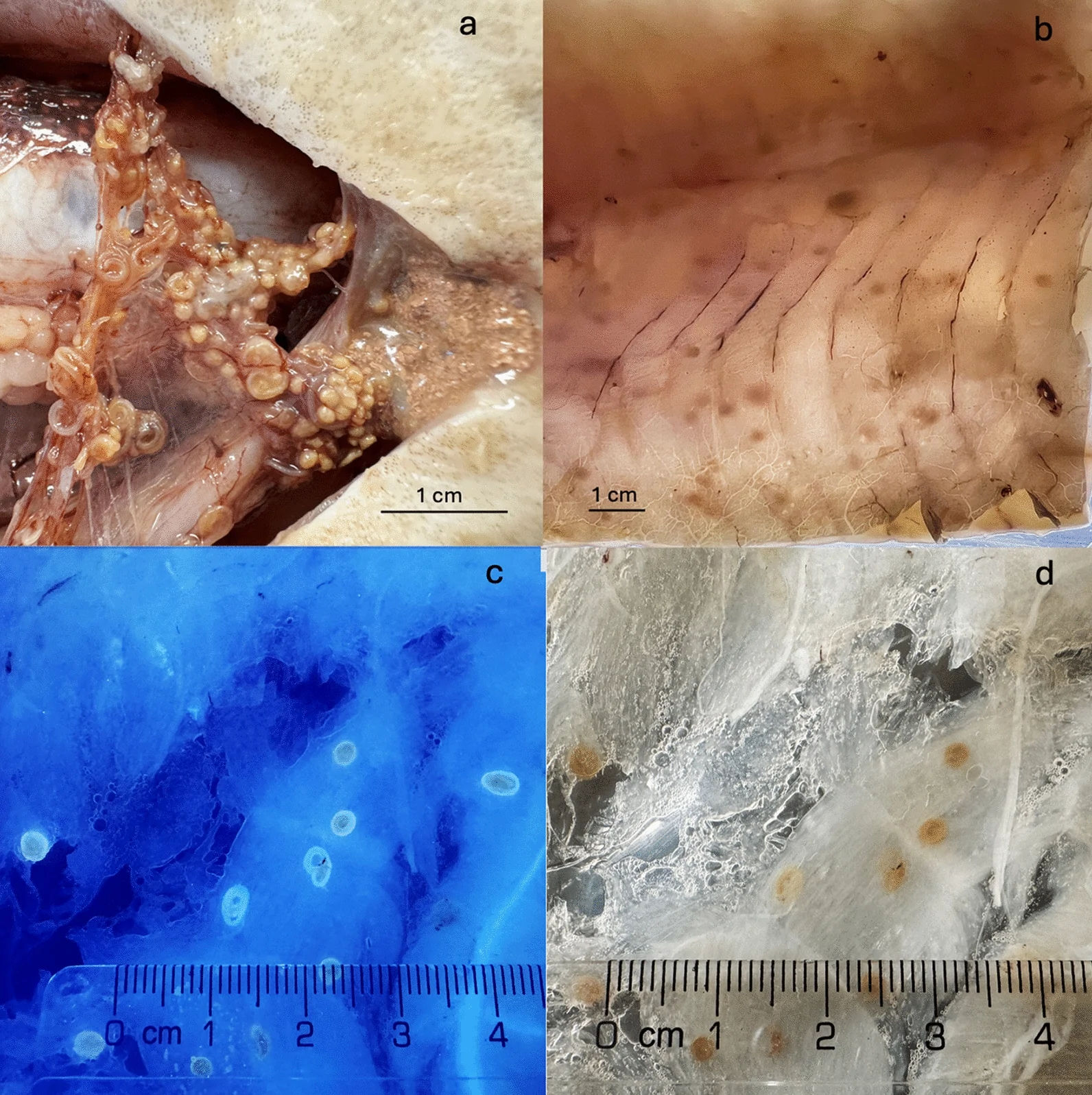
**a**–**d** Close-up views of *Anisakis simplex* (s.s.) larvae infecting tusk: **a** cluster of *A. simplex* (s.s.) larvae in the fish’s viscera, near the rectum; **b** *A. simplex* (s.s.) larvae, visible as dark spots, infecting the anterior ventral musculature (belly flap); **c** *A. simplex* (s.s.) larvae visible in the pressed anterior ventral fish muscle visualized under UV light; **d** same portion of pressed fish fillet shown in “c” with visible *A. simplex* (s.s.) larvae under natural light on a transparent glass

Over 27,000 anisakid nematode larvae were detected in 187 tusk specimens. The majority of larvae recovered from the skeletal muscle (i.e. flesh), liver, and visceral organs (including the digestive tract and gonads) belonged to the genus *Anisakis*, representing approximately 96% of all recovered nematodes. More than 600 larvae (2.5%) found in the skeletal muscle and liver exhibited morphological characteristics of *Phocanema*, including a prominent caecum extending alongside the ventriculus, and were often larger than the other nematodes [18, 26]. A total of 372 larvae (1.5%) from the surface of the digestive tract and liver were assigned to the genus *Contracaecum* (s.l.) larval type. In addition, 170 adult specimens of the raphidascaridid *Hysterothylacium* (s.l.) were found in the digestive tract of tusk. Some unidentifiable nematodes were recovered from cysts containing degraded worms, found in the viscera of a tusk caught off Frøya Island and in the belly flaps of a single fish from Skagerrak (Fig. 3a).

Data of the *cox*2 mtDNA sequences (580 bp) obtained from the analysed nematodes are reported in Table 2. Partial *cox*2 mtDNA sequences (580 bp) obtained from 52 *Anisakis* type I larvae specimens showed 100% identity with *Anisakis simplex* (s.s.) sequences deposited in GenBank (accession number MN961159). Of the 141 *Phocanema* specimens genetically identified, 51 *cox*2 sequences matched (> 99.47%) *Phocanema decipiens* (s.s.) (accession no. OP418116), 79 matched (99.47%) *Phocanema krabbei* (accession no. OP418118), and 11 matched (100%) *P. bulbosum* (accession no. OP418114). Out of the 30 *cox*2 sequences obtained from *Contracaecum*-type larvae, 29 matched (98.94%) *Contracaecum osculatum* B (accession no. OP418113), whereas one sequence from the Bear Island sample matched (99.36%) *Phocascaris cystophorae* (accession no. EU477209).

**Table 2:** Number of ascaridoid nematode larvae identified by mtDNA *cox*2 sequencing in *Brosme brosme* across the six sampling locations

	Bear Island	Nordkapp Bank	Vestfjord	Off Frøya	Off Bergen	Skagerrak	Total
*Anisakis simplex* (s.s.)	6	10	2	2	21	11	52
*Phocanema krabbei*	*–*	26	12	34	6	1	79
*Phocanema decipiens* (s.s.)	*–*	27	1	5	3	15	51
*Phocanema bulbosum*	11	–	–	–	–	–	11
*Phocascaris cystophorae*	1	–	–	–	–	–	1
*Contracaecum osculatum* B	8	21	–	–	–	–	29
*Hysterothylacium aduncum*	–	–	–	1	–	–	1
*Hysterothylacium* sp.	–	–	–	1	–	–	1
Total	26	84	15	43	30	27	225

Values represent total counts of each species per area, with overall totals reported in the last column

A single sequence with *Contracaecum*-like morphology from the Nordkapp Bank matched over 98.41% with *C. osculatum* from *Reinhardtius hippoglossoides* from Greenland waters (accession no. PV975769), and 96.42% to *Phocascaris cystophorae* from the harp seal (*Pagophilus groenlandicus*) from Newfoundland (accession no. EU477209). Based on sequence alignment with the sequences here obtained, this specimen showed higher similarity to the *Phocascaris cystophorae* than to *Contracaecum*, and was therefore reported here as *Phocascaris* sp. A *cox*2 sequence obtained from an adult specimen of *Hysterothylacium* matched 99.82% with *Hysterothylacium aduncum* from *Pollachius virens* from the Barents Sea (accession no. MW14907). A *cox*2 sequence obtained from an unidentified nematode recovered from a cyst containing degraded specimens in the viscera of a tusk caught from off Frøya Island, showed < 85% similarity to *Hysterothylacium deardorffoverstreetorum* from *Genypterus brasiliensis* in Brazil (accession no. MF276917).

 The mtDNA cox2 sequences of *Anisakis*, *Phocanema*, *Contracaecum*, *Phocascaris*, *Hysterothylacium*, and the unidentified nematode were deposited in GenBank under the following accession numbers: PZ565020 (*Anisakis simplex* (s.s.), Bear Island); PZ548206 (*Anisakis simplex* (s.s.), Finnmark), PZ548207 (*A. simplex* (s.s.), Vestfjorden), PZ548208 (*A. simplex* (s.s.), Off Frøya Isl.), PZ548209 (*A. simplex* (s.s.), Off Bergen), PZ548210 (*A. simplex* (s.s.), Skagerrak); PZ548201 (*Phocanema krabbei*, Finnmark), PZ548202 (*P. krabbei*, Vestfjorden), PZ548203 (*P. krabbei*, Off Frøya Isl.), PZ548204 (*P. krabbei*, Off Bergen), PZ548205 (*P. krabbei*, Skagerrak); PZ548196 (*Phocanema decipiens *(s.s.), Finnmark), PZ548197 (*P. decipiens* (s.s.), Vestfjorden), PZ548198 (*P. decipiens* (s.s.), Off Frøya Isl.), PZ548199 (*P. decipiens *(s.s.), Off Bergen), PZ548200 (*P. decipiens *(s.s.), Skagerrak); PZ565019 (*P. bulbosum*, Bear Island); PZ548195 (*Contracaecum osculatum* sp. B, Finnmark); PZ548213 (*Phocascaris* sp., Finnmark); PZ548214 (*Phocascaris cytophorae*, Bear Island); PZ548211 (*Hysterothylacium aduncum*, Finnmark); and PZ548212 (*Hysterothylacium* sp., Off Frøya Isl.).

### Anisakid infection characteristics

**Fig. 4:**
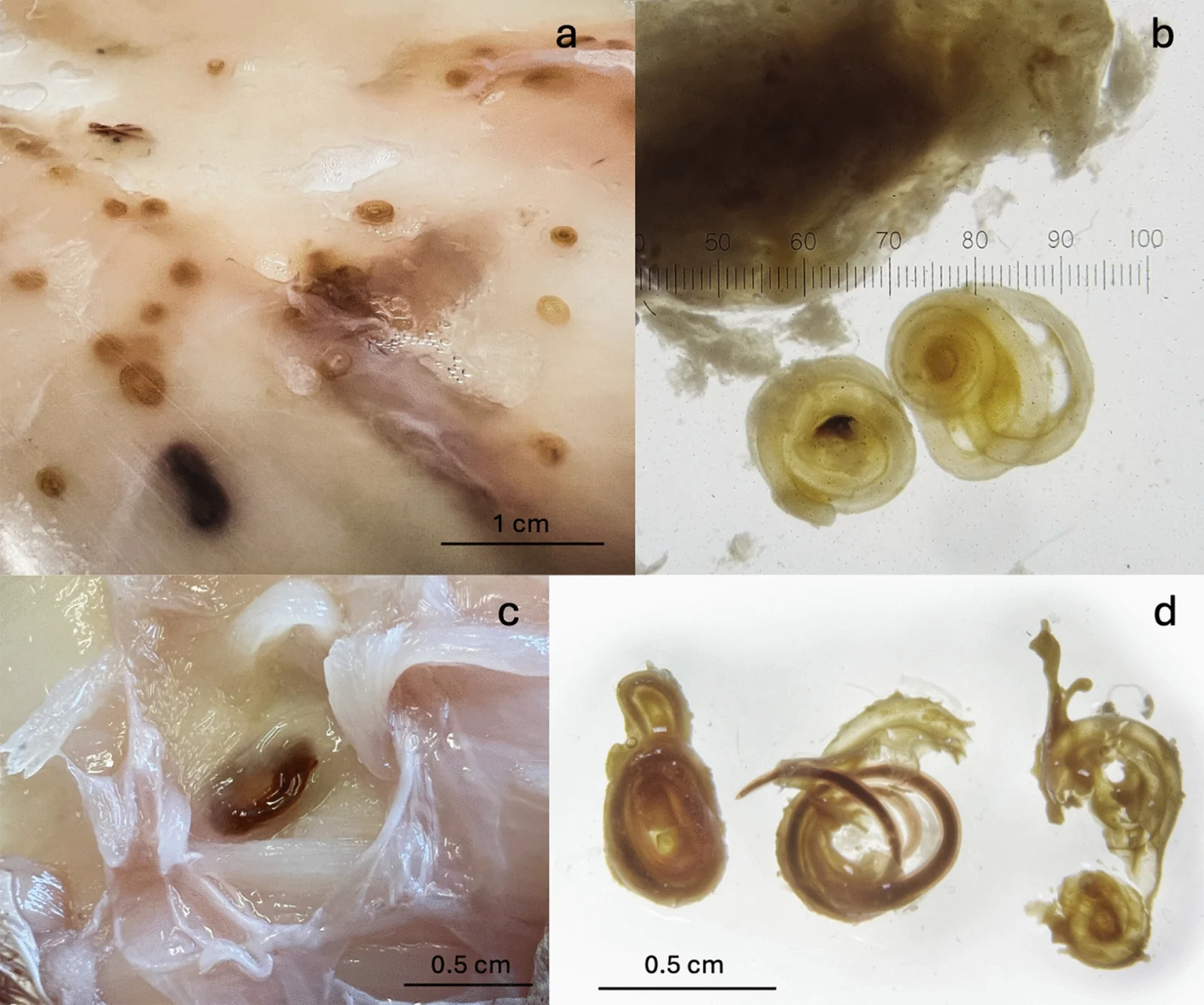
**a**–**d** Close-up views of *A. simplex* (s.s.) larvae **a–b** and *P. decipiens* (s.l.) **c**–**d** infecting tusk: **a** *A. simplex* (s.s.) larvae visible in the pressed anterior ventral musculature; **b** *A. simplex* (s.s.) larvae under the microscope, showing a well-defined thick capsule; **c*** P. decipiens* (s.l.) larva visible in the anterior ventral musculature; **d*** P. decipiens* (s.l.) larvae under the microscope: from left to right a larva enclosed in a thick capsule, an opened capsule with the larva partially emerging, and the residual capsule tissue

*Anisakis simplex* (s.s.) larvae detected in the musculature (flesh) were typically coiled and deeply encapsulated in thick, dark tissue (Fig. 3a–d and 4a, b; Table 3), and were often visible by naked eye. In some cases, strong encapsulation impaired larval fluorescence under UV light, making detection more difficult. Larvae located in the visceral cavity of the fish frequently occurred in clusters attached to the visceral organs, most commonly in the rectum area (Fig. 3a). Occasionally, necrotic mesenteric tissue was observed in the rectum area, apparently associated with clusters of *Anisakis* larvae and the cysts containing the unidentified nematode (Fig. 3a).

**Table 3:** Infection parameters of *Anisakis simplex* (s.s.) in *Brosme brosme* across the six sampling areas, reported separately for visceral organs and muscle. Prevalence (*P*, %), mean abundance (*A*), and mean intensity (MI ± SD; range in parentheses) are provided, together with total number of larvae (*N*) and their relative proportion (%)

			Viscera		Muscle				Total
			Liver	Rest of viscera	Viscera total	Bellyflaps	Flesh	Flesh total	
Bear Island	*P* (%)		100	100	100	100	86.7	100	100
	*A*		16.7	43.4	54.1	58.7	2.0	60.7	114.8
	mI (± SD) (min–max)		16.7 ± 10.2 (1–54)	43.4 ± 36.5 (2–135)	54.1 ± 44.8 (5–199)	58.7 ± 33.1 (6–133)	2.3 ± 1.2 (0–5)	60.7 ± 33.7 (8–138)	114.8 ± 69.9 (13–285)
	*N* (%)		320 (9.3%)	1303 (37.8%)	1623 -	1762 (51.2%)	59 (1.7%)	182 -	344 -
	Density (N/100g)		16.6	–	–	–	–	9.6	6.9
Nordkapp Ban	*P* (%)		100	100	100	100	86.2	100	100
	A		15.8	80.3	96.1	59.5	3.6	63.1	159.3
	mI (± SD) (min–max)		15.8 ± 9.8 (1–37)	80.3 ± 48.6 (20–220)	96.1 ± 53.8 (29–244)	59.5 ± 18.4 (28–110)	4.2 ± 6.1 (0–29)	63.1 ± 22.3 (31–139)	159.3 ± 66.9 (60–353)
	*N* (%)		465 (9.7%)	2394 (50.1%)	2859 -	1816 (37.9%)	106 (2.2%)	1922 -	4781 -
	Density (*N*/100g)		16.7	–	–	–	–	9.6	10.9
Vestfjorden	*P* (%)		93.5	100	100	100	41.4	100	100
	*A*		17.4	92.7	110.1	50.1	4.5	54.2	163.9
	mI (± SD) (min–max)		18.6 ± 12.2 (0–43)	92.7 ± 81.9 (7–264)	110.1 ± 90.5 (11–298)	50.1 ± 32.1 (10–124)	10.8 ± 8.7 (0–25)	54.2 ± 35.6 (10–144)	163.9 ± 119.9 (22–403)
	*N* (%)		576 (11.1%)	2902 (55.7%)	346 -	1598 (30.7%)	130 (2.5%)	1728 -	5208 -
	Density (N/100g)		26.7	–	–	–	–	6.5	8.38
Off Frøya	*P* (%)		87.5	100	100	100	93.8	100	100
	*A*		10.7	67.2	77.9	46.3	13.3	59.6	137.5
	mI (± SD) (min–max)		12.2 ± 15.3 (0–73)	67.2 ± 85.5 (1–326)	77.9x ± 97.5 (1–380)	46.3 ± 40.1 (5–188)	14.2 ± 22.8 (0–124)	59.6 ± 53.9 (7–217)	137.5 ± 143.3 (8–597)
	*N* (%)		345 (7.8%)	2158 (48.7%)	2503	1499 (33.8%)	426 (9.6%)	1925	4428
		Density (*N*/100g)	12.1	–	–	–	–	7.1	6.7
Off Bergen		*P* (%)	83.9	100	100	100	87.1	100	100
		*A*	15.6	75.5	91.2	60.9	5.2	66.1	157.2
		mI (± SD) (min—max)	18.6 ± 19.1 (0–66)	75.5 ± 89.9 (2–420)	91.2 ± 99.9 (2–468)	60.9 ± 56.1 (2–214)	5.9 ± 5.7 (0–26)	66.1 ± 59.8 (2–224)	157.2 ± 149.3 (4–692)
		*N* (%)	497 (9.9%)	2398 (48.2%)	2895 -	1920 (38.6%)	163 (3.3%)	2083 -	4978 -
		Density (*N*/100g)	7.5	–	–	–	–	3.4	3.35
Skagerrak		*P* (%)	100	100	100	100	43.3	100	100
		*A*	17.1	52.0	69.0	60.7	2.8	63.5	132.5
		mI (± SD) (min—max)	17.1 ± 13.7 (1–61)	52.0 ± 43.1 (3–145)	69.0x ± 49.1 (7–162)	60.7 ± 39.4 (6–159)	6.5 ± 5.3 (0–20)	63.5 ± 41.2 (6–164)	132.5 ± 75.2 (14–248)
		*N* (%)	516 (12.9%)	1568 (39.1%)	2084 -	1837 (45.9%)	84 (2.1%)	1921	4005
		Density (*N*/100g)	20.4	–	–	–	–	2.8	3.6

Larval density (number of larvae per 100 g of tissue) is also reported where applicable

**Fig. 5:**
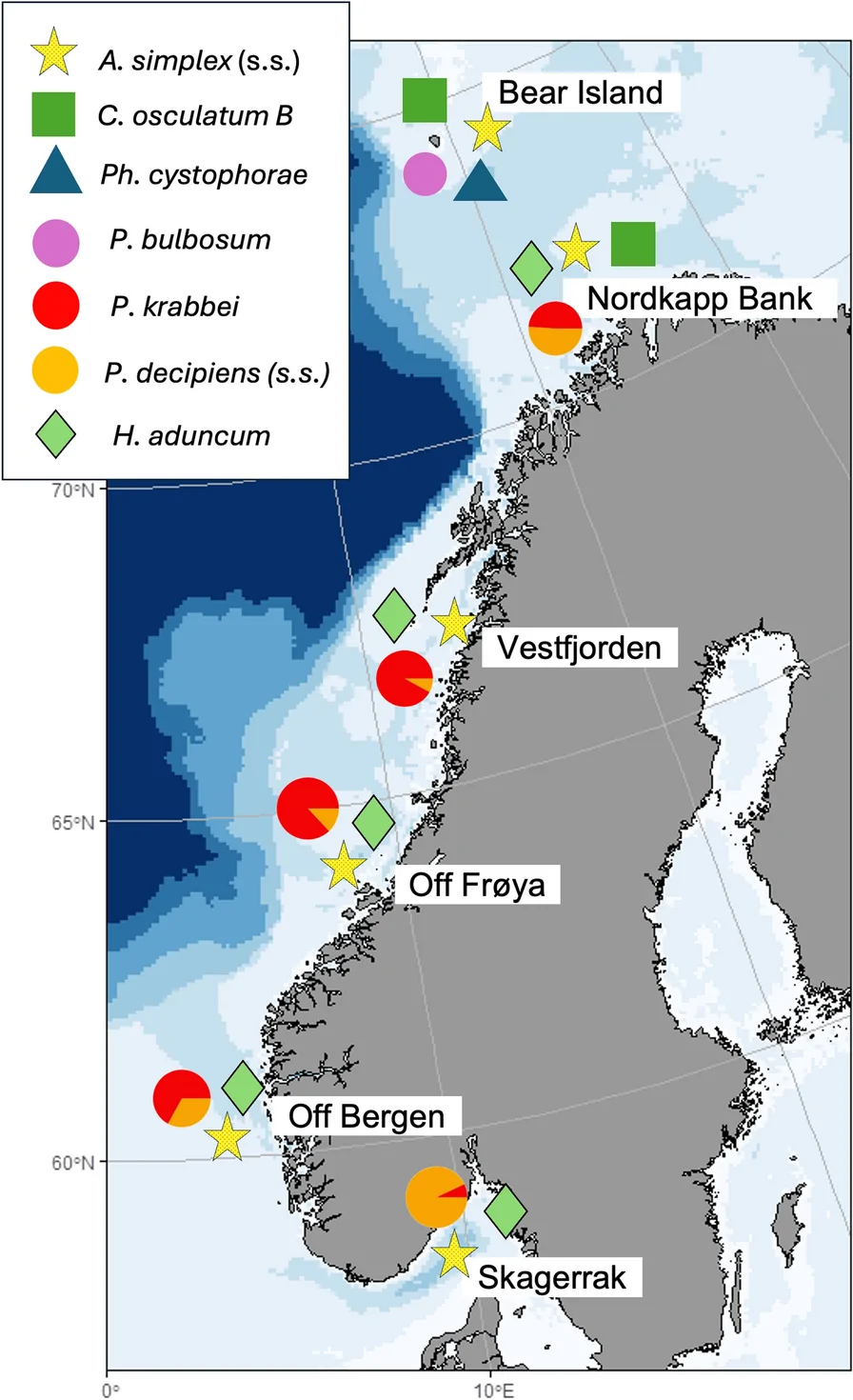
Spatial distribution of anisakid and raphidascarid parasite species in tusk from the investigated areas of the Northeast Atlantic

All *P. decipiens* (s.s.) and *P. krabbei* larvae were found in the fish musculature, where they were typically strongly encapsulated (Fig. 4c, d and Table 4). Based on the genetically identified subsample, the relative proportions of these two species varied among fishing areas (Table 2). In tusk samples from Nordkapp Bank, the two species occurred in approximately equal proportions (50%). In samples from Vestfjorden and off Frøya Island, *P. krabbei* was predominant, representing 90% and 87% of the identified larvae, respectively, whereas *P. decipiens* (s.s.) accounted for 10% and 13%. In tusk caught off Bergen, *P. krabbei* comprised approximately 70% of the identified larvae. In the Skagerrak sample, *P. krabbei* was also dominant, representing about 90% of the identified *Phocanema* larvae (Fig. 5).

**Table 4:** Infection parameters of *Phocanema s.l.* in *Brosme brosme* across the six sampling areas, reported separately for visceral organs and muscle

		Liver	Bellyflaps	Flesh	Total
Bear Island	*P* (%)	20	0	0	20
	*A*	0.3	0	0	0.3
	mI (± SD) (min—max)	1.7 ± 1.0 (1–3)	0 ± 0 0–0)	0 ± 0 (0–0)	1.7 ± 1.0 (1–3)
	*N* (%)	10 (100%)	0	0	10
	Density (*N*/100g)	–	0	–	0.02
Nordkapp Bank	*P* (%)	0	72.4	55.2	90
	*A*	0	2.4	1.6	4.0
	mI (± SD) (min—max)	0 ± 0 (0–0)	3.3 ± 2.3 (1–8)	2.9 ± 2.4 (1–10)	4.5 ± 3.4 (1–18)
	*N* (%)	0	72 (60.0%)	49 (40.0%)	121
	Density (N/100g)	–	0.6	–	0.3
Vestfjorden	*P* (%)	0	74.2	35.5	75
	*A*	0	3.5	1.7	5.2
	mI (± SD) (min—max)	0 ± 0 (0–0)	4.7 ± 6.6 (1–33)	4.8 ± 6.1 (1–22)	6.9 ± 10.7 (1–55)
	*N* (%)	–0	112 (67.9%)	53 (32.1%)	165
	Density (*N*/100g)	–	0.6	–	0.3
Off Frøya	*P* (%)	0	81.2	71.9	88
	*A*	0	6.1	3.4	9.4
	mI (± SD) (min—max)	0 ± 0 (0–0)	7.5 ± 7.3 (1–29)	4.8 ± 3.3 (1–12)	10.7 ± 9.7 (1–37)
	*N* (%)	0	196 (63.2%)	114 (36.8%)	310
	Density (N/100g)	–	1.2	–	1.2
Off Bergen	*P* (%)	0	15.2	3.2	21
	*A*	0	0.2	0.06	0.3
	mI (± SD) (min—max)	0 ± 0 (0–0)	1.2 ± 0.5 (1–2)	2.0 ± NA (1–2)	1.4 ± 0.5 (1–2)
	*N* (%)	0	7 (70.0%)	3 (30.0%)	10
	Density (N/100g)	–	0.02	–	0.01
Skagerrak	*P* (%)	0	50.0	33.3	64.5
	*A*	0	1.0	0.6	1.6
	mI (± SD) (min—max)	0 ± 0 (0–0)	2.0 ± 1.2 (1–4)	1.9 ± 0.7 (1–3)	2.5 ± 1.5 (1–6)
	*N* (%)	0	30 (61.2%)	19 (38.8%)	49
	Density (*N*/100g)	–	0.08	–	0.04

Prevalence (*P*, %), mean abundance (*A*), and mean intensity (MI ± SD; range in parentheses) are provided, together with total number of larvae (*N*) and their relative proportion (%). Larval density (number of larvae per 100 g of tissue) is also reported where applicable

In the Bear Island sample, the *Phocanema* specimens were identified as *P. bulbosum,* all occurring in the fish liver (Table 4). *Contracaecum osculatum* B occurred exclusively on pyloric caeca and intestine of tusk from Nordkapp Bank and Bear Island (Table 4). Based on genetic analyses, one specimen of *Phocascaris cystophorae* was detected in the viscera of a tusk caught off Bear Island, and one specimen of *Phocascaris* sp. in the viscera of a tusk from the Nordkapp Bank. Adult *Hysterothylacium aduncum* were detected in the stomach and intestine of tusk from all sampling locations, except the Bear Island (Table 6). Marked size variability of *Anisakis* and *Phocanema* L3 larvae was observed in both musculature and visceral tissues (Figs. 3, 4).

The main infection descriptors for each parasite genus and infection site are presented in Tables 3 and 6. The prevalence of *A. simplex* (s.s.) larvae was 100% in both viscera and musculature in all tusk samples (Table 3), whereas infections by *Phocanema* spp. and *Contracaecum* spp. varied among sampling areas (Tables 4 and 5).

**Table 5:** Infection parameters of *Contracaecum osculatum* B in *Brosme brosme* from Bear Island and Nordkapp Bank

		Liver	Viscera	Total
Bear Island	*P* (%)	30	0	30
	*A*	0, 5	0	0, 5
	mI (± SD) (min–max)	1.6 ± 0.9 (1–3)	0 ± 0 (0–0)	1.6 ± 0.9 (1–3)
	*N* (%)	14 (100%)	0	14
	Density (*N*/100g)	0,3	–	0, 03
Nordkapp Bank	*P* (%)	83,3	46,7	90
	*A*	10, 5	1, 5	11, 9
	mI (± SD) (min–max)	12.5 ± 11.3 (2–50)	3.1 ± 1.8 (1–8)	13.3 ± 11.9 (1–50)
	*N* (%)	314 (87.7%)	44 (12.3%)	358
	Density (N/100g)	15, 7	–	0, 8

Prevalence (*P*, %), mean abundance (*A*), and mean intensity (MI ± SD; range in parentheses) are given, together with total number of larvae (*N*) and their relative proportion (%). Larval density is expressed as number of larvae per 100 g of tissue

**Fig. 6:**
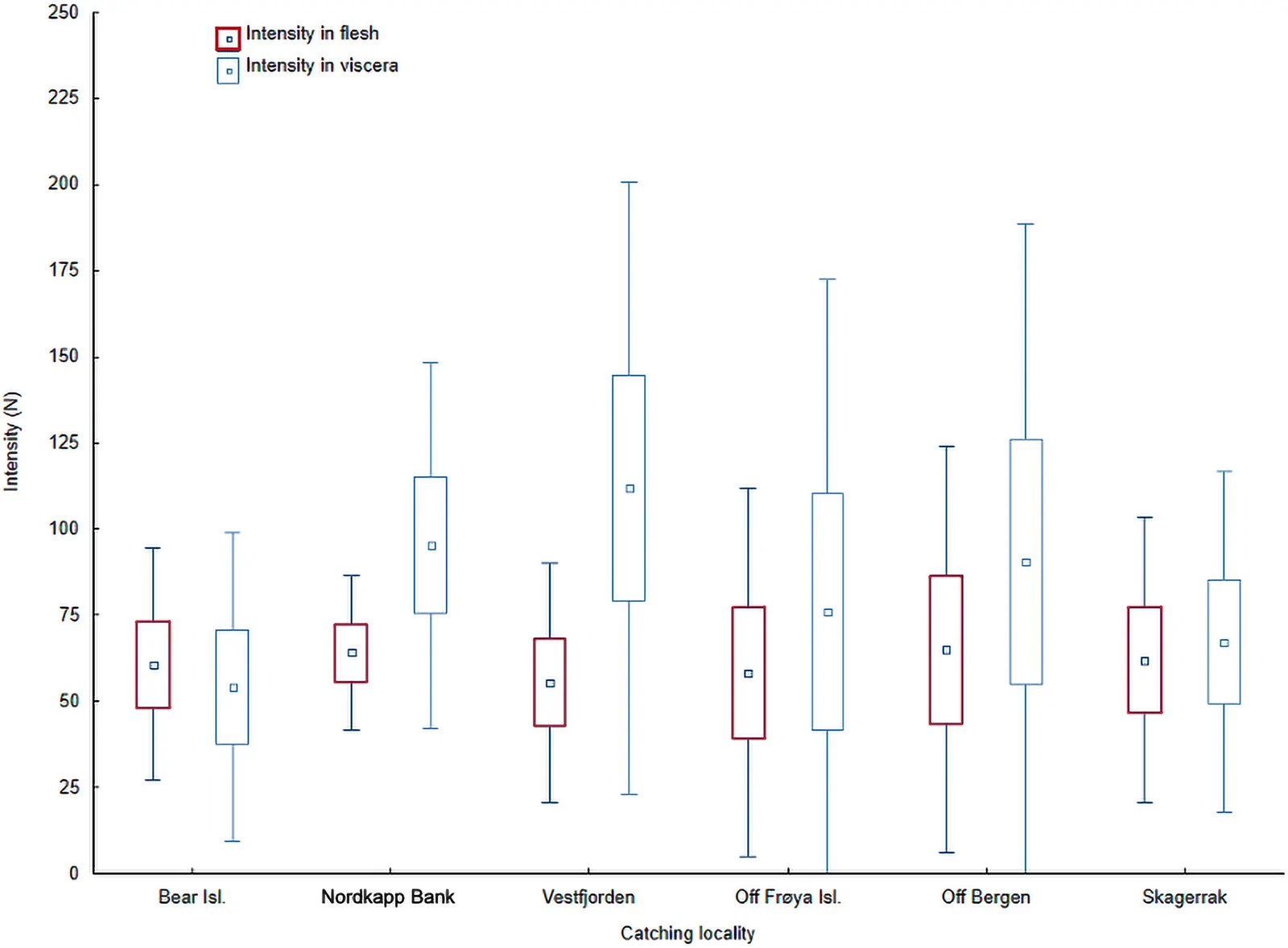
Overall intensity and intensity in the flesh of *A. simplex* (s.s.) larvae, by catching locality of tusk

Mean intensity of *A. simplex* (s.s.) both overall and in the fish musculature (intensity equals abundance at 100% prevalence) did not differ significantly among sampling localities (Kruskal–Wallis ANOVA, followed by Post hoc multiple comparisons of significance; *p* > 0.05) (Table 3 and Fig. 6).

GLM-analysis showed that tusk body weight had a slightly stronger effect on *Anisakis* infection probability than catch locality, although both factors were significant (LOG (TW + 1): *χ*^*2*^ = 57.6, *p* < 0.001; locality: *χ*^*2*^ = 49.9; *p* < 0.001). However, the effects of locality and host size could not be fully separated due to the lack of overlap in tusk body weight between the northern and central, and the southern samples (Fig. 2).

**Fig. 7:**
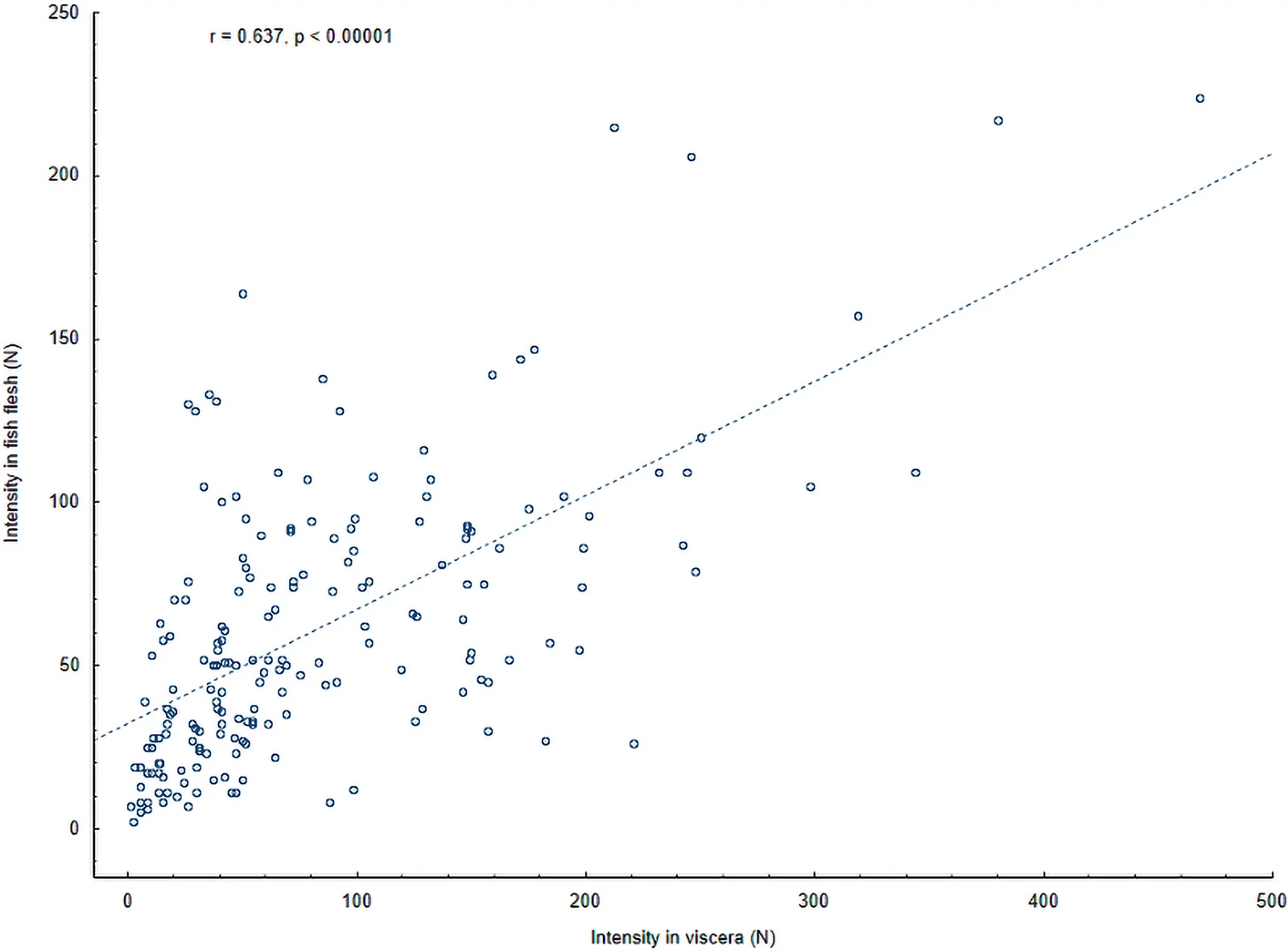
*A. simplex* (s.s.) intensity in the viscera against intensity in the fish flesh

A strong significantly positive correlation was observed between larval intensity in the viscera and larval intensity in the musculature (*r* = 0.637, *p* < 0.0001) (Fig. 7). Fish harbouring more than 100 larvae around the visceral organs typically also contained at least 25 larvae in the musculature.

**Fig. 8:**
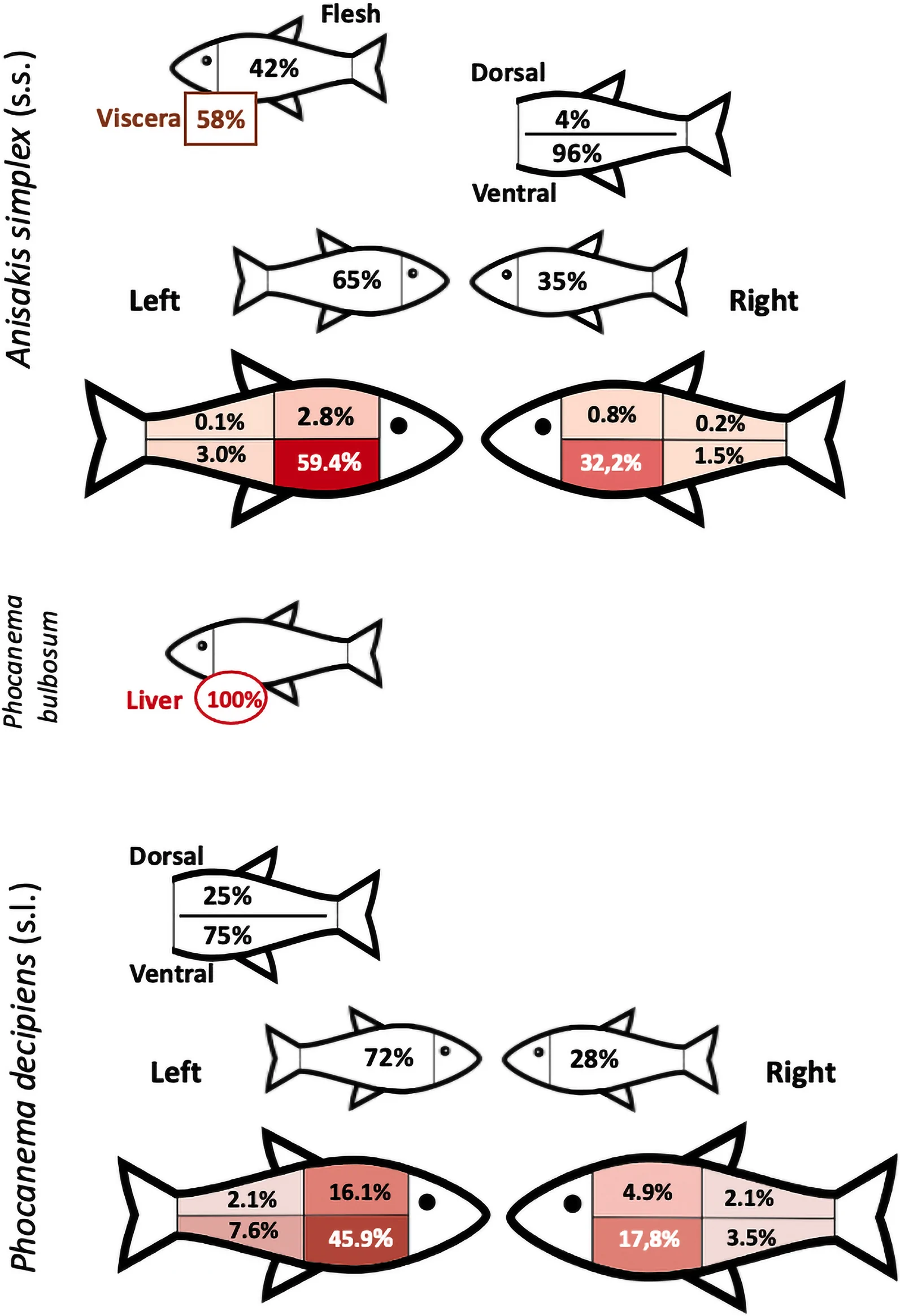
Localization and relative frequencies of *A. simplex* (s.s) and *P. decipiens* (s.l.) in the fish tissues of tusk. Colours are graded from dark red to white based on the % values

**Fig. 9:**
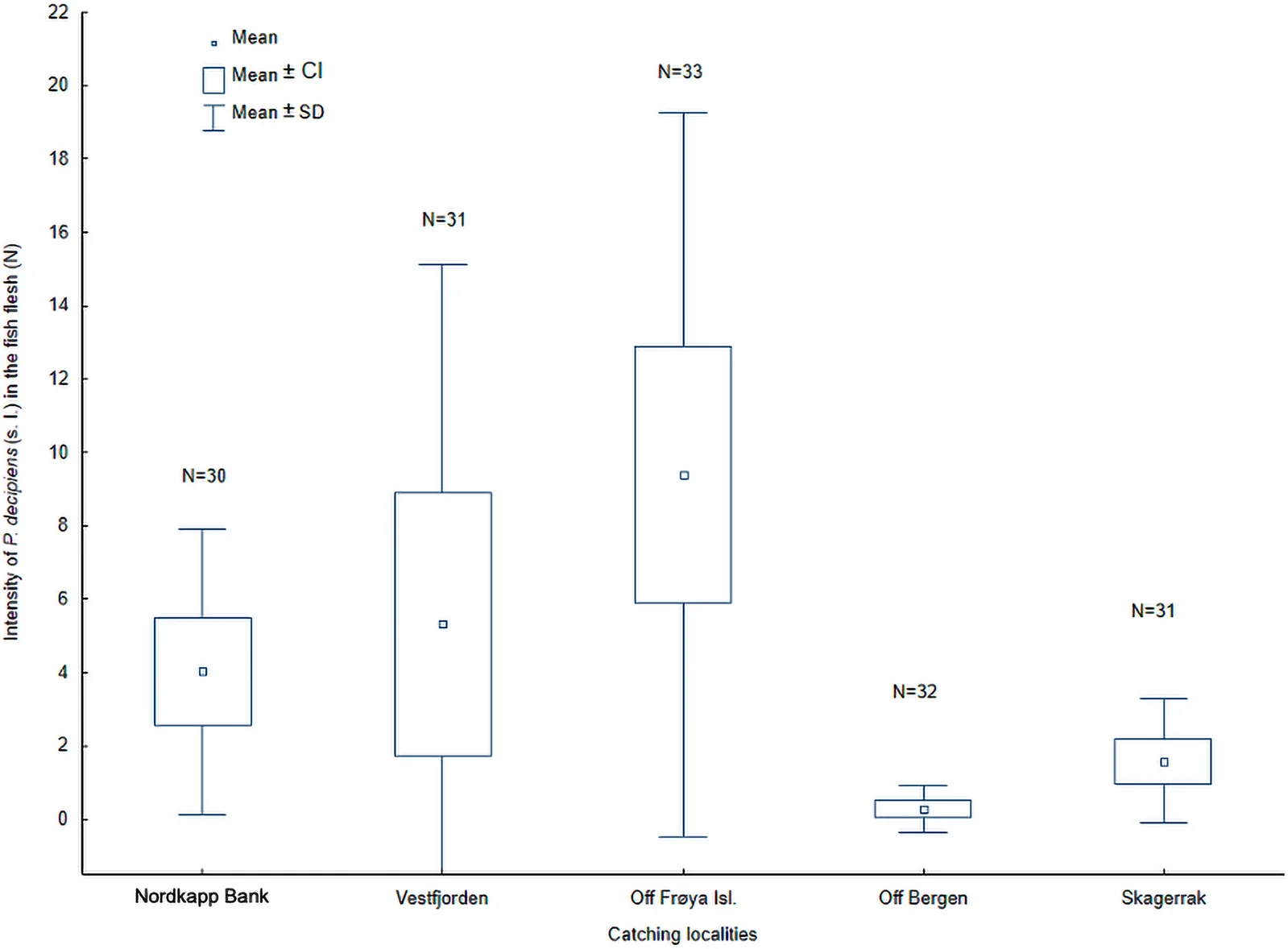
Intensity of *P. decipiens* (s.l.) larvae in the flesh by catching locality of tusk

*Anisakis simplex* (s.s.) larvae were unevenly distributed within the host. Overall, 58% of the larvae occurred in the viscera, whereas 42% were detected in the musculature (Fig. 8). The detailed distribution pattern is shown in Fig. 9. Among the *A. simplex* larvae located in the viscera, 40% were embedded in the stomach wall musculature, 30% occurred on the intestine, pylorus, and mesenteries, 17% in the liver, and 4% on the gonads. Within the musculature, the majority of *A. simplex* (s.s.) larvae (90.5%) occurred in the anterior ventral section (belly flap) of each side fillet, whereas only 9.5% were located in the dorsal and caudal regions of the fillet (both dorsal and ventral) (Fig. 8). *A. simplex* (s.s.) larvae in the musculature were also unevenly distributed between fillet sides, with 65% located in the left fillet and 35% in the right side.

The overall infection levels of *P. decipiens* (s.s.) and *P. krabbei* in the musculature differed significantly among samples (Fig. 8 and Table 4), with highest values recorded in tusk caught off Frøya Island, and lowest in samples from the North Sea off Bergen. Most larvae (64%) were located in the anterior ventral musculature (bellyflap) (Fig. 8) and their distribution between the flesh sides was uneven, with 72% occurring in the left side and 28% in the right side. In tusk from Bear Island, all *P. bulbosum* specimens occurred in the liver and at low intensities (Table 4 and Fig. 8).

GLM-analysis revealed that catching locality of tusk had a considerably stronger effect on infection probability of *P. decipiens* (s.l.) larvae compared to (log) fish host body weight (Locality: *χ*^*2*^ = 101.7; *p* < 0.00001; LOG (TW + 1): *χ*^*2*^ = 35.6, *p* < 0.0001). This pattern was mainly explained by the significantly lower intensity of *P. decipiens* (s.l.) in the generally larger tusks from the two southern sampling localities.

*Contracaecum osculatum* larvae occurred at a relatively high abundance in the viscera of tusk from Nordkapp Bank, where they were located on the pyloric caeca and encysted over the intestine. In contrast, only low numbers were found over the intestine of fish from the Bear Island sample (Table 5).

Infection levels of adult *Hysterothylacium aduncum* varied among regions, with the highest values recorded in tusk from Skagerrak (*P* = 38.7%, *A* = 1.4) and the lowest in the Vestfjorden sample (*P* = 12.5%, *A* = 0.4), whereas this parasite was not detected in the Bear Island sample (Table 6).

**Table 6:** Infection parameters of *Hysterothylacium aduncum* in *Brosme brosme* across the six sampling areas, based on specimens recovered from the stomach

		Stomach
Bear Island	*P* (%)	0
	*A*	0
	mI (± SD) (min–max)	0 ± 0 (0–0)
	*N*	0
Nordkapp Bank	*P* (%)	33.3
	*A*	1.8
	mI (± SD) (min–max)	5.4 ± 4.7 (2–15)
	*N*	54
Vestfjord	*P* (%)	12.5
	*A*	0.4
	mI (± SD) (min–max)	3.3 ± 3.3 (1–8)
	*N*	13
Off Frøya	*P* (%)	21.2
	*A*	0.8
	mI (± SD) (min–max)	3.7 ± 3.0 (1–10)
	*N*	26
Off Bergen	*P* (%)	32.3
	*A*	1.0
	mI (± SD) (min–max)	3.2 ± 2.4 (1–7)
	*N*	35
Skagerrak	*P* (%)	38.7
	*A*	1.4
	mI (± SD) (min–max)	3.6 ± 3.7 (1–12)
	*N*	43

Prevalence (*P*, %), mean abundance (*A*), mean intensity (MI ± SD; range in parentheses), and total number of individuals (*N*) are reported

## Discussion

This study represents the first systematic investigation of nematodes infecting tusk, collected from six areas along the Norwegian continental shelf. The present tusks, primarily consisting of large, mature individuals characterized by opportunistic feeding in deep waters of the continental shelf and slope, hosted a wide diversity of nematode parasites, including nine ascaridoid species. Considering that most previous studies on tusk did not employ genetic methods for parasite species identification, apart from sporadic reports on *A. simplex* (s.s.) [20, 21, 24], all other species documented here represent the first specific records of these taxa in this fish species. The systematic approach applied to samples from multiple areas enabled the characterization of general infection patterns, including geographic variation across fishing grounds, and revealed consistent differences in parasite assemblages as well as clear preferences of the different nematode taxa for specific anatomical sites within the host. Such differences in parasite assemblages indirectly reflect parasite life cycles, tusk feeding ecology and structuring, and the distribution of definitive hosts, and therefore provide ecological information on parasite transmission pathways, host feeding ecology, and host–parasite interactions in Norwegian marine ecosystems. *A. simplex* (s.s.) was present in all tusks examined, infecting muscle, liver, gonads, and viscera. Over 50% of the worms found in fish viscera were embedded in the stomach wall of the fish. It is unknown whether these larvae represented transient individuals migrating into the visceral lumen or were blocked in the stomach wall tissue. *Anisakis simplex* (s.s.) appeared to be relatively evenly distributed across the different sampling batches (Fig. 5). It appears that beyond a certain size threshold (around 50 cm), tusk show relatively stable infection intensities, as also reported by Hauksson [23]. Consequently, fish body size does not appear to be a reliable predictor of *A. simplex* (s.s.) larval infection levels in commercial size tusk from Norwegian fishing grounds. This pattern contrasts with that observed in other gadiforms, such as Arctic cod and European hake (*Merluccius merluccius*) [30–32], in which positive correlations between fish size and anisakid burden are commonly reported. Such correlations are generally attributed to the progressive accumulation of larvae over the host’s lifespan, assuming that anisakid larvae can persist in host tissues for extended periods.

*Phocanema* and *Contracaecum* species exhibited marked differences in distribution across catching localities, indicating a geographically structured infection pattern across sampling areas likely reflecting ecological variation and differences in the availability of suitable intermediate and/or definitive hosts (Fig. 8 and Tables 4 and 5). *Phocanema* infection was present in all tusk samples examined, but the different species identified showed distinct geographic distributions. *Phocanema decipiens* (s.s.) and *P. krabbei* were detected in the muscle tissue of all samples, except for tusk from Bear Island where only *P. bulbosum* was identified from liver (Figs. 5 and 9, Tables 2 and 5). The two *Phocanema* species found in muscle tissue, *P. decipiens* (s.s.) and *P. krabbei*, occurred more frequently in the dorsal and caudal musculature than *Anisakis* (Fig. 8). The ability of *Phocanema* spp. to migrate into the dorsal musculature has been previously documented not only in tusk [23] but also in several other fish host species including Northeast Arctic cod and European hake [31, 32]. As observed for *A. simplex* (s.s.), a left-side preference was also recorded for *P. decipiens* (s.l.), with over 70% of larvae located on the left flesh side (Fig. 8).

*Contracaecum osculatum* B was the only *Contracaecum* species found in tusk. Larvae were present in the viscera, on the liver and in the digestive tract, and occurred exclusively in the northernmost sampling areas, namely Nordkapp Bank and Bear Island (Table 5). Two additional anisakid species utilizing pinnipeds as definitive hosts were identified at low infection levels in the viscera of tusk from Arctic waters. *Phocascaris cystophorae* was detected in a one tusk from Bear Island, whereas an unidentified *Phocascaris* sp. was recovered from a fish sampled at Nordkapp Bank.

In addition to anisakid nematodes, adult specimens of the raphidascaridid *Hysterothylacium* (s.l.) were detected in the digestive tracts of tusk from all sampling locations, except for Bear Island. Morphological and genetic analyses identified these adult nematodes as *H. aduncum*. Infection levels by *H. aduncum* in tusk were relatively low, with prevalence ranging from 12% in Vestfjorden to 38% in Skagerrak, and absence in the Bear Island sample. These values are markedly lower than those reported for *H. aduncum* in cod and saithe from Nordkapp Bank, where prevalence ranged between 81 and 100%, with a strong seasonal effect and higher infection levels in winter and early spring [25]. The present tusk samples from the Nordkapp Bank which were also collected in winter, showed a prevalence of 33% and mean abundance of 1.8, considerably lower than the 100% prevalence and mean abundance of 80 reported for cod from the same area and season [25]. These differences in infection levels may reflect ecological differences between the two fish species, including depth distribution and feeding habits.

Previous studies reporting ascaridoid infections in tusk from the North Atlantic are few and often based on limited sample size but are generally consistent with the patterns observed in the present study. High prevalence of *A. simplex* (s.l.) has been repeatedly reported in tusk from Norwegian and North Atlantic waters [18, 19, 23], supporting the consistently high infection levels observed here across all sampling areas. [18] reported 100% prevalence of *A. simplex* (s.l.) in tusk from southwestern Norway, together with the presence of *Phocanema* (reported as *Pseudoterranova* s.l.) in the musculature and *Hysterothylacium aduncum* and *Contracaecum* sp. in the viscera, while [19] found similarly high prevalence of *A. simplex* (s.l.) and occurrence of *H. aduncum* in tusk collected from deep waters off the Faroe Islands.

In addition, *A. simplex* (s.s.) larvae have been reported in tusk from the North Sea and other areas of the Northeast Atlantic, although infection levels were not specified [20, 21]. More extensive sampling from Icelandic waters also confirmed 100% prevalence of *A. simplex* (s.l.) in tusk muscle and the occurrence of *Pseudoterranova* (s.l.), with infection levels ranging within the same order of magnitude as those observed in the present study [23].

Records from other North Atlantic areas, including the North Sea and Gulf of Maine, likewise describe high anisakid infection levels in tusk, although comparisons are limited by differences in geographic area, sampling design, detection methods and sample size [22, 24]. In particular, [22] reported high abundance of *Phocanema* (reported as *Pseudoterranova*) in tusk from the Gulf of Maine, higher than those observed in the present study, which may reflect local ecological conditions and a stronger spatial overlap between tusk and seal definitive hosts.

### Ecological considerations

The distribution patterns of anisakid species infecting tusk in Norwegian waters appear to reflect differences in parasite life cycles and the ecology of their definitive hosts. *A*. *simplex* (s.s.) showed highly overlapping infection levels across all sampling areas, independent of fish host body size and spanning a wide latitudinal gradient from southern to northern Norwegian waters. Tusk appears to be a relatively sedentary species, showing limited large-scale movements between fishing banks and no clear evidence of seasonal inshore–offshore migrations. The species is thought to undertake mainly local depth-related movements within the same geographical area, shifting between deeper and shallower waters [2]. This relatively homogeneous pattern across distant fishing grounds and the sedentary habit of this species suggests a stable and widespread exposure to infective larvae throughout the present tusk sampling areas. This pattern may be linked both to the broad distribution and high mobility of cetaceans, which serve as definitive hosts for *A. simplex*, facilitating a widespread and stable availability of infective larvae in marine food webs, as well as the vagility of the wide trophic spectrum of prey species involved in the life cycle of this parasite. Considering that tusk is generally regarded a non- to moderately migratory demersal species, the similar infection levels of *A. simplex* over distant areas with different ecological characteristics suggest that larval exposure and recruitment is relatively stable across fishing grounds. Moreover, this pattern suggests that tusk reaches a maximum infection level for *A. simplex* larvae, with larval turnover driven by host-induced mortality through immunological means along with continuous reinfection through predation.

Differently, the geographically structured infection patterns observed for *Phocanema* spp. and *Contracecum* are consistent with the more restricted distribution ranges and definitive host specificity of these parasites, which rely primarily on pinnipeds to develop into adults and reproduce [16, 33]. *Phocanema* species composition showed a clear spatial pattern: *P. decipiens* (s.s.) and *P. krabbei* occurred across multiple fishing grounds along the Norwegian coast, from Skagerrak to the Nordkapp Bank, although the relative proportions varied (Fig. 5). These species are mainly associated with common seals (*Phoca vitulina*) and grey seals (*Halichoerus grypus*) as definitive hosts [33], both widely distributed along the Norwegian coastline, which may explain the broad occurrence of these parasites in tusk. In contrast, *P. bulbosum* was found exclusively in tusk caught in the Bear Island area, where the other *Phocanema* species were absent. This area falls within the distribution range of the bearded seals (*Erignathus barbatus*) [34], which is considered among the preferred definitive hosts for *P. bulbosum* [16].

Notably, *C*. *osculatum* B and *Phocascaris cystophorae* were also found exclusively in tusk from Arctic waters. For instance, the occurrence of *Ph*. *cystophorae* appears to be consistent with the distribution range of its definitive host, the hooded seal (*Cystophora cristata*) [35, 36]. Similarly, the abundant presence of harp seal (*Pagophilus groenlandicus*) in the Barents Sea can explain the finding of *C. osculatum* B, which seems to act as definitive host this anisakid species [16]. Overall, the spatially structured anisakid assemblages observed in tusk likely reflect the population structure, distribution, and movement patterns of marine mammals in Norwegian coastal and offshore ecosystems, resulting in localized differences in parasite exposure and infection pressure.

The present Barents Sea tusk samples exhibited notably low infection levels with *C. osculatum* B, in stark contrast to other commercially important whitefish species such as cod, saithe, and haddock caught in the same fishing grounds (Nordkapp Bank) [31]. In these areas, cod and saithe showed a prevalence of 100%, with parasite abundances ranging from 35 to 180 worms per fish, while 80% of haddock specimens were infected, albeit at lower intensities (mean abundance of 6 worms per fish) [31]. The comparatively low infection rates observed in tusk (Table 5), despite being caught in the same waters as these heavily infected species, suggest species-specific differences in parasite exposure or establishment success. Such differences may be related to ecological traits of the host, including diet composition, habitat use, depth distribution, and trophic niche. Tusk is a demersal predator typically associated with deep shelf and slope habitats, and its feeding ecology differs from that of cod, saithe, and haddock, which may result in reduced exposure to infective stages of *Contracaecum*. Alternatively, the observed differences may reflect different host–parasite interactions, potentially involving species-specific responses of the fish host to parasite establishment. Notably, in cod, saithe, and haddock, *C. osculatum* B larvae are known to preferentially aggregate in the pyloric caeca [31]. Whether the comparatively low infection levels observed in tusk are related to reduced exposure, lower establishment success, or differences in tissue tropism of the parasite remains unclear and warrants further investigation. Interestingly, *Contracaecum* larvae were completely absent in all tusk samples from Vestfjorden to Skagerrak, despite the presence in these waters of several *Contracaecum* species commonly reported from other gadiform fishes, such as European hake, cod, and other demersal species [17, 30, 32]. This absence further suggests that infection patterns in tusk are strongly influenced by species-specific ecological and trophic characteristics, differentiating its fish ecology from the other gadiform.

According to population genetic studies, tusk show low but significant genetic structuring across the Northeast Atlantic [37]. Large-scale larval drift and limited movements of demersal juveniles and adults among neighbouring sites have been suggested as possible sources of gene flow between localities in the North Atlantic [37]. However, there is no evidence that tusk perform extensive migrations after reaching a demersal mode of life [1, 2, 38]. The fish species appears to be largely stationary as adult and is also characterized by widespread spawning rather than discrete spawning aggregations [2, 37, 39, 40].

In this context, the distinct nematode assemblages presently recorded in tusk, which likely reflect the distribution of their marine mammal definitive hosts, support the view that this species is largely non-migratory and exhibits some degree of population structuring. While *A. simplex* (s.s.) typically accumulates over the lifespan of larger predatory fish throughout the NE Atlantic [30, 31], the species composition and infection levels of *Phocanema* and *Contracaecum* seem to reflect that distinct ecological conditions prevail in several of the present catching localities of tusk. For example, the nematode species profile in tusk caught off Bear Island, characterized by *P. bulbosum* as the only *Phocanema* species present, strongly suggests that actual fish have not been exposed to *P. decipiens* (s.l.) larvae which are closely interlinked with coastal seal populations. Thus, tusk from the central Barents Sea seem to belong to a distinct population which members are largely confined to this area and apparently separated from their siblings in the southern Barents Sea and below. Consequently, parasite assemblages may provide useful biological clues of population structure and geographic separation among tusk stocks in the Northeast Atlantic.

To further evaluate this hypothesis, targeted investigations on parasitic nematodes, based on a broader set of genetic identifications and fine scale population genetic analyses, combined with fish host population genetics, otolith microchemistry, as well as life history traits, would help to better define population boundaries and assess the potential of parasites as biological indicators for stock discrimination.

Tusk is a top demersal predator characterized by a predominantly benthic feeding strategy, relying largely on bottom-dwelling prey. This species typically inhabits deep waters and exhibits relatively low vagility, exploiting benthic resources along offshore continental shelf and slope habitats. It is generally described as a demersal generalist feeder, with crustaceans and polychaetes consistently reported as dominant prey items, while fish constitute a comparatively minor component of the diet [1, 4, 41, 42] investigated the ecological characteristics and diet of tusk in the Northwest Atlantic and reported a more piscivorous and opportunistic feeding behaviour compared to other studies. In contrast, more recent investigations from the Northeast Atlantic have refined this view, indicating that tusk predominantly feeds on benthic decapods, particularly *Munida* spp. and *Lithodes maja*, whereas fish and polychaetes represent secondary prey items [43, 44]. Notably, [44] reported that only four out of more than 140 examined individuals had consumed fish (i.e. unidentified pleuronectids, *Sebastes* spp., and *Clupea harengus*). The possible role of benthic decapod crustaceans in anisakid transmission to tusk remains to be investigated. However, the parasite assemblages observed in the present study suggest that tusk may have a more piscivorous feeding behaviour than generally assumed.

Infection levels of *A. simplex* (s.s.) in tusk were high (mean abundance: 143), and similar to those reported for more piscivorous gadiform species including adult and commercial sized Northeast Atlantic cod (mean abundance: 125) and saithe (mean abundance: 130) [30, 31, 45]. Such similarity in parasite burdens is not straightforward to explain, given the different feeding ecology usually attributed to these species. One possible explanation is that fish prey contributes more substantially to the diet of tusk than suggested by stomach content studies, at least in larger individuals. *Anisakis simplex* (s.s.) is typically transmitted through pelagic or benthopelagic prey, and benthic crustaceans are not considered common intermediate or paratenic hosts of this parasite. The high proportion of larvae embedded in the stomach wall, often representing more than half of the visceral parasites, may reflect recent ingestion of heavily infected prey, possibly fish. The lower but persistent presence of adult *H. aduncum* in the digestive tract supports the hypothesis of continuous intake of infected fish prey, since this parasite at adult stage tends to complete development and reproduce within a limited time frame [25], in contrast to anisakid larvae that may persist for years in paratenic hosts.

An alternative explanation is that the peculiar *A. simplex* (s.s.) infection pattern observed in tusk, characterized by larvae deeply embedded in muscle tissue and encapsulated within unusually thick capsules (Figs. 3 and 4), reflects long persistence of these larvae within host tissues rather than repeated exposure events. In this study, tusk exhibited significantly higher abundances of *A. simplex* (s.s.) larvae in the musculature than cod or saithe, and levels comparable to those recorded in the highly piscivorous hake [32]. According to the limited data currently available, tusk appears to exhibit a slow growth rate, reaching sexual maturity at approximately 50 cm and 8–10 years of age, while individuals up to 70 cm in length may be as old as 20 years [46]. Gradually accumulated over the lifespan of the fish, some of these larvae may represent older individuals retained in host tissues for extended periods and that are not necessarily viable. More detailed investigations, including viability assessments and immunopathological studies examining the host’s inflammatory and immune responses to these larvae, would be necessary to clarify this hypothesis.

Interpretation of tusk feeding ecology based on stomach contents remains challenging. Tusk frequently exhibits empty or partially digested stomachs, likely reflecting long digestion times and intermittent feeding. In addition, capture-related stress, prolonged soak times of passive gears, and barotrauma during ascent may cause stomach eversion and regurgitation, leading to the loss of stomach contents prior to sampling [47]. Together, these factors bias stomach content analyses, particularly in larger and deeper-caught individuals, and may result in an underestimation of prey diversity and feeding intensity [48, 49]. Overall, the parasite assemblages observed in tusk provide ecological information that complements traditional diet studies, suggesting that this species may exploit a broader trophic spectrum than previously assumed and highlighting the value of parasites as indicators of host feeding ecology and trophic interactions.

### Food safety and quality implications

Given the zoonotic nature of most anisakid species identified, a special focus was placed on assessing their presence in the edible part of the fish. The overall density of *Anisakis simplex* (s.s.) larvae per 100 g of fish muscle (N/100 g in Table 3) differed significantly among sampling areas, ranging from a maximum of 9.6 larvae/100 g in samples from the Nordkapp Bank and Bear Island to 2.8 larvae/100 g in samples from the Skagerrak. These values were calculated based on the total weight of the filleted muscle, although 92% of the larvae were localized in the belly flap region. This raises concern regarding the zoonotic and allergenic risks associated with larval density in this muscle portion, which is still commonly marketed as an edible product.

Tusk exhibited high infection levels in the flesh, notably higher (mean abundance = 61 larvae) than those reported for Northeast Arctic cod (*Gadus morhua*, mean abundance = 13 larvae), haddock (*Melanogrammus aeglefinus* Linnaeus, 1758; mean abundance = 3 larvae), and saithe (*Pollachius virens* Linnaeus, 1758; mean abundance = 18 larvae) from the Northeast Atlantic [30, 31, 46], although lower than those recorded in European hake (*Merluccius merluccius*, mean abundance = 75 larvae) [32]. Compliance with freezing or cooking recommendations issued by major food safety authorities, such as the European Food Safety Authority (EFSA) and the U.S. Food and Drug Administration (FDA), effectively reduces the risk of zoonotic transmission [50–52] demonstrated that these freezing treatment recommendations should be applied with caution and an adequate safety margin, as the suggested temperatures must be reached at the core of the fish to ensure the complete inactivation of parasite larvae. This aspect may be particularly relevant in domestic settings, where home freezers may not always achieve sufficiently low or stable temperatures, or when fish are stored in large frozen blocks that can impede uniform freezing.

In addition to these preventive measures, the present results suggest that specific processing strategies may further reduce consumer exposure. In particular, trimming the belly flaps of tusk could remove over 90% of the parasite burden. Accordingly, limiting commercialization to dorsal and caudal fillets, where larval densities are consistently lower, may represent an effective approach to mitigate both anisakiasis and allergenic risks.”

This processing approach has previously been recommended for European hake (*Merluccius merluccius*) with heavy *Anisakis* infections [53–55], as well as for other fish species known to accumulate larvae in muscle tissues adjacent to the visceral cavity [15, 31, 56, 57]. However, such trimming measures are less effective for *Phocanema* larvae, which show generally higher abundances in the dorsal and caudal musculature of their fish hosts (Fig. 8 and Table 4), thereby maintaining a potential risk for consumers even in apparently cleaner fillet portions. *Phocanema* larvae in tusk muscle and liver tissues may reach 4–5 cm body length and are often reddish or brownish in colour. These characteristics enhance their visibility in fish fillets, and some larvae can be readily detected with the naked eye (Fig. 4).

In addition to the food safety implications, the presence of anisakid larvae in the musculature and viscera may also represent a relevant food quality issue. Larvae recovered from tusk (*Brosme brosme*) from all catching localities frequently exhibited a peculiar condition. In the fish flesh, both *A. simplex* (s.s.) and *Phocanema* spp. larvae were encapsulated in thick, hardened brownish capsules (Fig. 4b, d), which made the larvae visible within the fish musculature even to the naked eye, while sometimes impairing their fluorescence under UV light. In some freshly examined fish, a proportion of these larvae remained viable after capsule removal, while others appeared partially degraded or desiccated (Fig. 4). In the fish visceral lumen, and particularly in the rectal area, *A. simplex* larvae tended to form large clusters embedded in thick, strong capsules, with many worms found dead or decomposed inside these structures (Fig. 3a).

The frequent visibility of larvae in both the visceral lumen and musculature means that they can be detected during routine quality inspections using a standard candling table (Fig. 3b), a procedure commonly employed in fish processing plants to identify parasites or other defects in fillets. Such visible infections may negatively affect product acceptability and commercial value, even when larvae are non-viable or removed during processing.

## Conclusions

Tusk is an increasingly important demersal species for commercial fisheries in Norwegian waters, yet its parasite fauna, feeding ecology and population structure remain poorly documented. The present study provides the first systematic description of ascaridoid nematode assemblages in tusk across multiple Norwegian fishing grounds, generating baseline data relevant for understanding parasite ecology and host–parasite interactions in this species. The infection patterns observed, particularly the consistently high abundance of *Anisakis simplex* (s.s.) across all sampling areas, indicate a stable and widespread exposure to anisakid larvae over a broad latitudinal gradient. This homogeneous pattern suggests that transmission is driven by large-scale ecological processes, including the wide distribution of cetacean definitive hosts and the trophic links connecting pelagic and demersal food webs. In contrast, seal-associated anisakids showed geographically structured distributions, reflecting the spatial ecology of pinniped hosts and localized differences in parasite transmission pathways, and limited migration patterns of tusk.

In addition, the parasite assemblages observed provide indirect information on the feeding ecology of tusk. High infection levels of *A. simplex* comparable to those observed in more piscivorous gadiform species suggest that fish prey may contribute more to the diet of tusk than previously assumed.

The high infection levels observed in the musculature, particularly in the belly flap region, may have implications for food safety and quality and consumer acceptance. Knowledge of parasite distribution within the host may therefore support the development of targeted processing strategies aimed at reducing parasite burden in commercial products.

Overall, integration of parasite data with dietary analyses, host genetics, and broader geographic sampling is recommended to better understand the ecology, population structure, and exploitation potential of tusk in Norwegian waters.

## Data Availability

All data generated or analysed during this study are included in this article. DNA sequences generated are deposited on GenBank under accession numbers reported in results, and available: https://www.ncbi.nlm.nih.gov/genbank/
